# When Prosthetic Valves Compete for Space: A Case of Transcatheter Aortic Valve Embolization Due to Prosthetic Mitral Valve

**DOI:** 10.7759/cureus.4299

**Published:** 2019-03-22

**Authors:** Arun K Nagabandi, Hemang Panchal, Rohit Srivastava, Nirat Beohar

**Affiliations:** 1 Interventional Cardiology, Mount Sinai Medical Center, Miami Beach, USA; 2 Interventional Cardiology, University of California San Francisco – Fresno Medical Education Center, Fresno, USA

**Keywords:** transcatheter aortic valve replacement, transcatheter aortic valve implantation, embolization, complication, prosthetic valves

## Abstract

Transcatheter aortic valve replacement (TAVR) is now the preferred choice of treatment for severe symptomatic aortic stenosis (AS) patients who are at intermediate to high risk for surgery. Rare complications like valve embolization have been described and we report a case with unique cause for such complication. A 79-year-old female presented with new onset dyspnea on exertion for evaluation and work up to the outside hospital and was found to have severe AS and referred to us for TAVR evaluation. She had a history of coronary artery bypass grafts surgery and bioprosthetic mitral valve replacement (MVR) 10 years ago. Preoperative transesophageal echocardiogram (TEE) revealed normally functioning bioprosthetic mitral valve and severe AS with peak/mean gradients of 67/44 mm Hg. She underwent transfemoral TAVR using a 26-mm Edwards Sapien S3 TAVR valve. During the slow deployment of the TAVR valve while rapid pacing, the valve appeared to move a little. Shortly after the removal of the delivery system out of the valve, the TAVR valve embolized to ascending aorta. It was carefully withdrawn into the aortic arch past the great vessels with an inflated balloon aortic valvuloplasty (BAV) catheter. Then, BAV was performed x 2 to plan for TAVR with a second valve, but the BAV balloon water-melon seeded repeatedly. We concluded that in this case, the rigid struts of bioprosthetic mitral valve encroaching on LVOT resulted in TAVR valve embolization and a decision was made to abort further attempts at TAVR valve implantation. This patient later under surgical aortic valve replacement (SAVR) and is clinically doing well at six months of clinical follow-up.

## Introduction

Transcatheter aortic valve replacement (TAVR) is now the preferred choice of treatment for patients with severe symptomatic aortic stenosis (AS) who are intermediate- to high-risk candidates for surgical aortic valve replacement (SAVR). While being less invasive than surgery, TAVR procedure is still associated with a few complications. Most common complications are conduction system abnormalities requiring permanent pacemaker placement, access site bleeding and other local complications, acute kidney injury, stroke, coronary obstruction and paravalvular leak (PVL) or regurgitation. Less common complications like valve embolization have been described but are very rare, and hence there is a scarcity of data in how to prevent these from happening and when happened, how to best manage these patients. Since the original approval of TAVR for high-risk patients in 2011, the number of sites and operators performing TAVR has steadily increased at a pace faster than expected [1], likely due to fewer complications and patient preference for this procedure over SAVR. As this procedure is increasingly being adopted and performed by new operators and centers, it becomes more important than ever for the operators to be aware of rare complications and know the available options. 

## Case presentation

A 79-year-old female with a past medical history of coronary artery disease (CAD) and coronary artery bypass grafts (CABG), bioprosthetic mitral valve replacement (MVR), hypertension, hyperlipidemia, obstructive sleep apnea, and atrial fibrillation presented to the clinic for the evaluation of worsening dyspnea. She underwent transthoracic echocardiogram (TTE) that revealed mild left ventricular hypertrophy, interventricular septum about 1.4-cm thick and normal left ventricular ejection fraction. In addition, she was found to have severe AS at outside hospital, thought to be causing her symptoms and was referred to us for TAVR evaluation.

**Figure 1:**
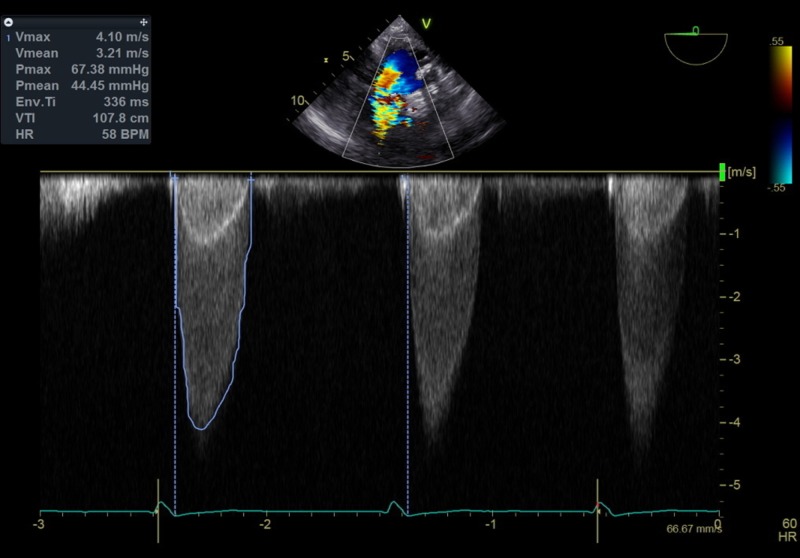
TEE image of continuous wave Doppler from deep transgastric view showing elevated velocities and gradients across the aortic valve TEE, transesophageal echocardiogram

After she underwent a comprehensive evaluation and reviewing her treatment options by the heart team, TAVR was recommended as preferred treatment. She had preprocedural computed tomography of chest/abdomen/pelvis for evaluation of access sites and for procedural planning. Her preprocedural transesophageal echocardiogram (TEE) revealed no thrombus in left atrial appendage and normally functioning mitral valve prosthesis and severe AS with peak/mean gradients of 67/44 mm Hg respectively, across her aortic valve (Figure 1).

She subsequently was scheduled for and underwent transfemoral TAVR using a 26-mm Edwards Sapien S3 TAVR valve. Bilateral femoral arterial access was obtained and TAVR valve prepared, aortic valve crossed with a wire in usual fashion and TAVR valve positioned across the native aortic valve without any difficulty. Rapid ventricular pacing was initiated and after confirming no loss of capture and no premature ventricular contractions (PVCs) with adequate reduction in systolic blood pressure to 45-50 mm Hg range, the valve inflation was done. During the slow inflation of balloon expandable TAVR valve, the valve appeared to move a little (Video 1).

**Video 1:** TAVR valve deployment while rapid pacing TAVR, transcatheter aortic valve replacement

Shortly after the withdrawal of valve delivery system and balloon into the descending thoracic aorta, the implanted TAVR valve embolized into ascending aorta. All the available options were thought of and it was carefully withdrawn into the aortic arch and positioned just after the great vessels with a inflated Edwards 25-mm balloon aortic valvuloplasty (BAV) catheter (Video 2).

**Video 2:** TAVR valve in aortic arch positioned after great vessels TAVR, transcatheter aortic valve replacement

Then, using the same Edwards 25-mm BAV catheter, valvuloplasty was performed x 2 to plan for second TAVR valve implantation but the BAV balloon water-melon seeded repeatedly during inflation (Video 3).

**Video 3:** BAV of aortic valve showing the water melon seeding of the balloon BAV, balloon aortic valvuloplasty

Alternate reasons for such occurrence like loss of pacing capture, PVCs or inadequate drop in blood pressure to <50 mm Hg were excluded. The intraprocedural TEE revealed normally functioning bioprosthetic mitral valve and thick valve struts encroaching on the non-calcified LVOT as shown (Video 4). 

**Video 4:** Intraprocedural TEE showing normally functioning bioprosthetic mitral valve and its proximity to aortic valve TEE, transesophageal echocardiogram

We concluded that the rigid struts of bioprosthetic mitral valve encroaching on the left ventricular outflow tract (LVOT) likely resulted in TAVR valve embolization and further attempts at TAVR valve implantation were aborted. She was discharged from hospital uneventfully and clinically followed and underwent SAVR two months after the index TAVR procedure with a 23-mm Carpentier-Edwards bovine pericardial tissue valve. At the time of SAVR, her TAVR valve in aortic arch was visualized and it had a freely mobile echodensity attached to the leaflet consistent with thrombus. She recovered well from surgery and discharged on warfarin anticoagulation. At clinical follow-up four months after the surgery, she was doing well with no other complications. 

## Discussion

TAVR valve embolization is a very rare complication of the procedure now-a-days, with no significantly adverse outcomes when managed effectively [2]. When TAVR valve embolizations do occur, most often they are into the aortic side than to the LVOT side [2-3], although numerous cases of ventricular embolizations have been recently reported [4-5]. Several causes of TAVR valve embolization were identified from earlier patient series of which mitral valve prosthesis is one of them [2-3]. A pre-existing mitral valve prosthesis can make the LVOT stiff and non-compliant due to the struts of the prosthetic mitral valve. Hypertrophic basal septum also mimics the same physiology, and both of these can be a cause of TAVR valve dislodgement and embolization after the procedure.

This potential complication can be identified and avoided by doing BAV beforehand with an appropriately sized balloon in patients with the mitral prosthesis. In cases where the balloon behaves unsteady during inflation, TAVR with a self-expanding CoreValve (ReValving Technology Medtronic Inc., Minneapolis, MN, USA) or SAVR might be a better alternative than a balloon expandable Edwards valve (Edwards Lifesciences, Irvine, CA, USA). Furthermore, these TAVR valves are reported to have subclinical valve or leaflet thrombosis and the likelihood of that in a position other than native aortic valve annulus is even higher [6-7]. When such valves embolize, it is always safer to try and re-position them after the great vessels of the arch (after left subclavian) to decrease the risk of stroke as illustrated in our case. 

## Conclusions

We concluded that in this case, the rigid struts of bioprosthetic mitral valve encroaching on LVOT resulted in TAVR valve embolization and advice caution in such situations. The operators should be aware of this very rare complication and know their options and try to avoid them. To our knowledge, apart from the very early case series that were published before the commercial use of TAVR valves for severe AS, no other cases identified the bioprosthetic mitral valve as one of the causes for TAVR valve malposition and embolization. Since the approval of TAVR valves and procedure in 2011 in United States, this has been a field with most research, most advances and innovations. We feel identifying and revisiting this potential cause of valve malposition and embolization is important and clinically relevant in this todays practice, as most of the patients with prior prosthetic mitral valve are considered at least intermediate risk for a SAVR procedure and are advised TAVR.
